# Comparison of CT values in traditional trajectory, traditional cortical bone trajectory, and modified cortical bone trajectory

**DOI:** 10.1186/s12893-022-01893-5

**Published:** 2022-12-27

**Authors:** Dongshan Liu, Alafate Kahaer, Yixi Wang, Rui Zhang, Abulikemu Maiaiti, Xieraili Maimaiti, Zhihao Zhou, Wenjie Shi, Zihao Cui, Tao Zhang, Longfei Li, Paerhati Rexiti

**Affiliations:** 1 Department of Spine Surgery, The First Affiliated Hospital of Xinjiang Medical University, Urumqi, 830054 Xinjiang Uygur Autonomous Region China; 2 Xinjiang Medical University, Urumqi, China; 3 Digital Orthopaedic Center, Xinjiang Medical University, Urumqi, China

**Keywords:** Pedicle screw, Cortical bone trajectory, Modified cortical bone trajectory, CT value, Zone of interest

## Abstract

**Background:**

To compare the CT values and length of the screw tracks of traditional trajectory (TT), cortical bone trajectory (CBT), and modified cortical bone trajectory (MCBT) screws and investigate the effects on the biomechanics of lumbar fixation.

**Methods:**

CT scan data of 60 L4 and L5 lumbar spine were retrieved and divided into 4 groups (10 male and 10 female cases in the 20–30 years old group and 20 male and 20 female cases in the 30–40 years old group). 3-dimentional (3D) model were established using Mimics 19.0 for each group and the placement of three techniques was simulated on the L4 and L5, and the part of the bone occupied by the screw track was set as the region of interest (ROI). The mean CT value and the actual length of the screw track were measured by Mimics 19.0.

**Results:**

The CT values of ROI for the three techniques were significantly different between the same gander in each age group (P < 0.05). The difference of screw track lengths for CBT and MCBT in the male and female is significant (P < 0.05).

**Conclusions:**

According to the CT values of the three screw tracks: MCBT > CBT > TT, the MCBT screw track has greater bone-screw surface strength and longer screw tracks than CBT, which is easier to reach the anterior column of the vertebral body contributing to superior biomechanical properties.

## Introduction

The traditional pedicle screw technique (TT) was first applied in 1963 by Roy-camille et al. [1] for the treatment of lumbar fractures, which has good biomechanical properties throughout the three columns of the spine and is widely used [2, 3]. In 2009, Santoni et al. proposed the cortical bone trajectory (CBT), which was designed to increase the contact between the screw and the cortical bone by changing the screw path and improving the stability [4, 5]. Recent studies have found that the CBT is more effective than the TT in improving mechanical stability, less intraoperative bleeding, shorter hospital stay, less intrusion of adjacent joints, and less paravertebral muscle damage, Based on these advantages, the CBT technique is no longer limited to osteoporotic patients and has been applied to bariatric patients and revision surgery [3, 6]. Authors found that the CBT technique still has shortcomings, the screw track does not utilize the medial and inferior wall of the pedicle and the bone cortex of the lateral edge of the upper endplate, the screw may damage the facet joint and the adjacent intervertebral disc, the screw entry point and the lateral wall of the pedicle were prone to rupture, and the screw entry point lacks reliable anatomical reference structures. For this reason, the authors proposed a modified cortical bone trajectory (MCBT) technique [7–9].

In recent years, the use of finite element analysis has been considered as a convenient and reliable research method with reliable variables and realistic experimental data [10]. In our previous finite element studies [10–13], the biomechanical properties of MCBT were investigated and proved to be superior to CBT and TT [11–14]. CT value of the screw path was thought to be the main reason for this superiority. This study is the first preliminary investigation of the CT values and lengths of TT, CBT, and MCBT techniques, and their effects on the biomechanics of lumbar posterior fixation, and provides the theoretical basis for the clinical application of the MCBT technique.

## Materials and methods

### Acquisition of 3D reconstruction data

A total of 60 high-resolution CT scans of the lumbar spine (including L4 and L5) aged 20–40 years from January 2015 to January 2022 were retrieved from the imaging department of our hospital. A total of 4 groups were divided according to age (20–30 years, 30–40 years) and gender. Among them, 10 males and 10 females were selected from the 20 to 30 years old group, and 20 males and 20 females were selected from the 30–40 years old group, totaling 60 cases. Inclusion criteria: (1) age between 20 and 40 years old; (2) good skeletal development and basic symmetry of the left and right anatomy. Exclusion criteria: (1) congenital malformations and other lesions in the spine; (2) lumbar spine tumors, infections and other lesions with bone defects or destruction; (3) obvious underlying diseases causing osteodystrophy, osteoporotic changes, etc. High-resolution computed tomography (HRCT) data (AQUIRRON 16, PHILIPS, Netherlands) was used and the data were saved in DICOM format.

### Construction of the 3D surgical model

**Fig. 1:**
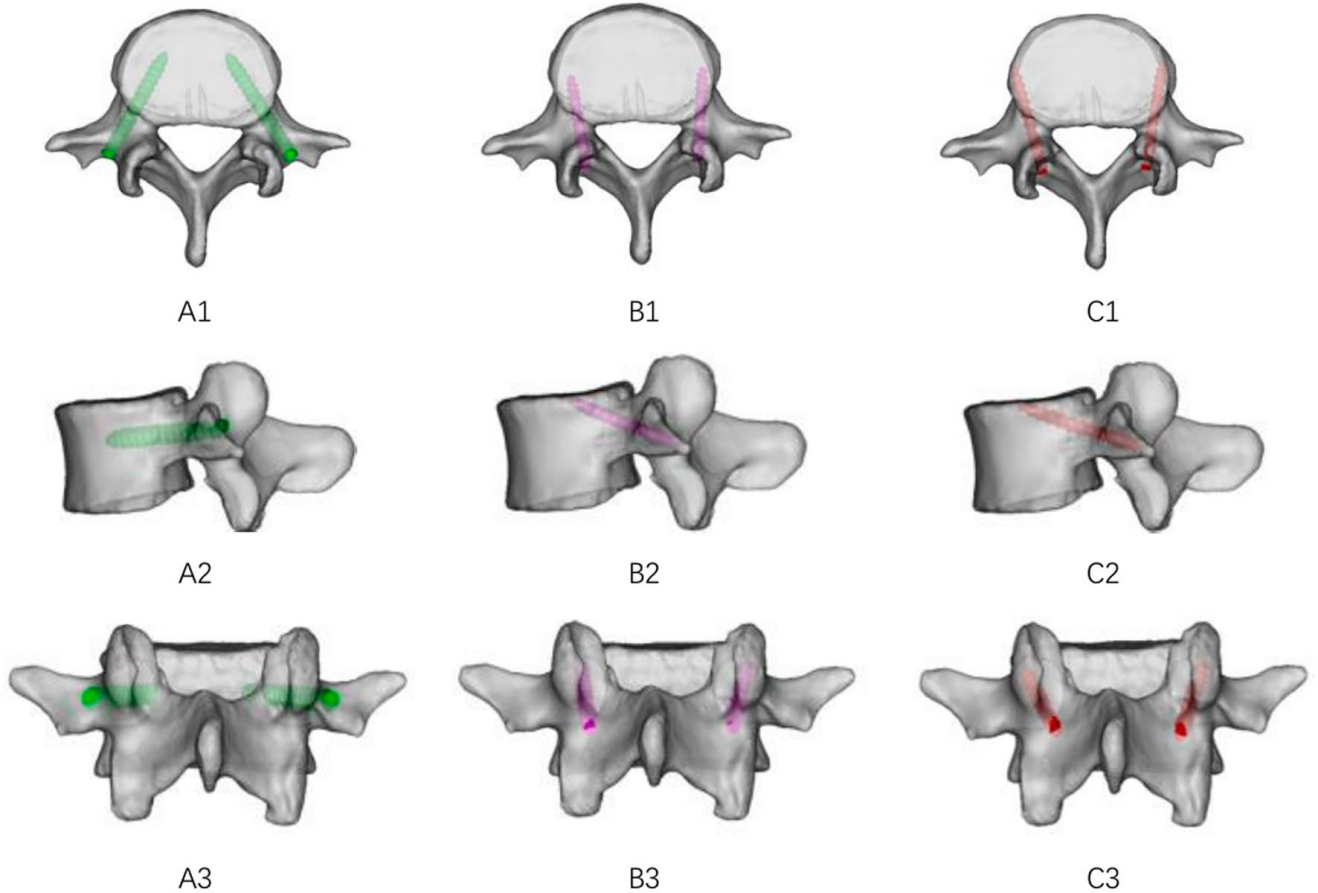
Model of the L4 vertebra and diagrams illustrating the trajectory from the axial and sagittal views. (**A 1–3**) TT screws at the L4; (**B 1–3**) CBT screws at the L4; (**C 1–3**) MCBT screws at the L4

**Fig. 2:**
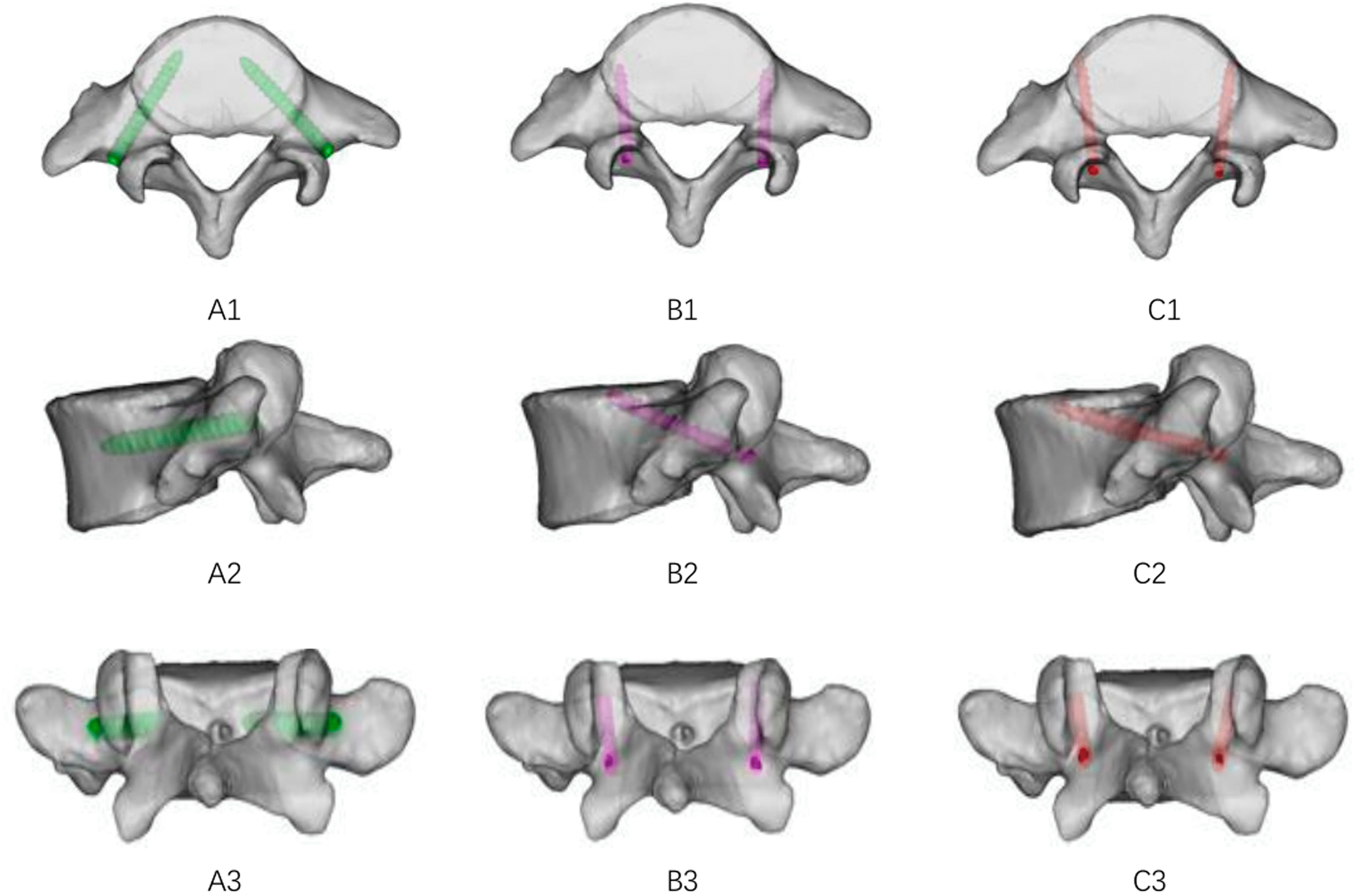
Model of the L5 vertebra and diagrams illustrating the trajectory from the axial and sagittal views. (**A 1–3**) TT screws at the L5; (**B 1–3**) CBT screws at the L5; (**C 1–3**) MCBT screws at the L5

The DICOM data from CT was imported into Mimics 19.0, and the bone tissue density window was selected to perform 3D reconstruction of L4 and L5. TT screw (6.0 mm in diameter and 45 mm in length), CBT screw (4.5 mm in diameter, 35 mm in length) and MCBT screw (4.5 mm in diameter, 40 mm in length) were reconstructed. The TT, CBT, and MCBT techniques were simulated on the L4 (Fig. 1) and L5 (Fig. 2).

With reference to the relevant literature, the TT were inserted at the intersection of the horizontal midline between the lateral edge of the superior articular process and the transverse process, with the screw path parallel to the superior endplate of the vertebral body [15]. The screw entry point for the CBT was the intersection of the vertical line at the center of the superior articular process and the horizontal line 1 mm below the inferior edge of the transverse process, with an abduction angle of approximately 10° and a head tilt angle of approximately 25–30° [16, 17]. The screw entry point for the MCBT was shifted 2.0–3.0 mm inward relative to the CBT technique, with a cephalic tilt angle of approximately 25° and a abduction angle of approximately 22° [7].

### Determination on the region of interest (ROI) and measures

The region of interest (ROI) was set as the portion of the bone occupied by each simulated screw track, representing the bone area of each tract. The ROIs were separated and the mean CT value of each ROI was measured using the corresponding analysis function of the software [18–20]. The actual length of the screw track was measured from the screw head to the part of the screw just outside the bone.

### Statistical analysis

SPSS 19.0 statistical software (SPSS, USA) was used for statistical analysis, and the mean CT values of ROI in each group were expressed as mean ± standard deviation (x ± s). The mean length and CT values of the ROI were compared between the L4 and L5 vertebrae in each age group of men and women with different techniques using the paired design data of t-test, and between the three techniques using the same paired design data of t-test. One-way ANOVA was used to compare the mean CT values and screw lengths of ROI for the same age group and different ganders for each technique. P < 0.05 was considered as statistically significant.

## Results

The mean CT values of the ROI around the screw tracks of the L4 and L5 vertebrae by age and gender are shown in Table 1, and the mean CT values of the ROI around the screw tracks of the L4 and L5 vertebrae by age and gender for TT, CBT, and MCBT were not statistically significant (P > 0.05), thus they were combined in each group. The mean CT values of the ROI around the screw tracks of different techniques for men and women in each age group are shown in Table 2, and they are statistically significant (P < 0.05) in the mean CT values of the ROI of MCBT, CBT and TT techniques between the same ganders in each age group. They was statistically significant (P < 0.05) in the mean CT values of ROI of MCBT compared with CBT in each group, and statistically significant (P < 0.05) in the mean length of MCBT and CBT trajectory (Table 3).

**Table 1:** The mean CT values within the ROI of both trajectories between L4 and L5 vertebral bodies was compared in male and female subjects at the same age

Age group	Traditional pedicle trajectory				Cortical bone trajectory				Modified cortical bone trajectory			
	Male		Female		Male		Female		Male		Female	
	L4	L5	L4	L5	L4	L5	L4	L5	L4	L5	L4	L5
21–30	258.8 ± 53.95	235.2 ± 64.2	339.3 ± 68.3	298.3 ± 67.7	447.3 ± 108.4	430.5 ± 96.7	535.4 ± 109.3	505.4 ± 104.8	511.0 ± 92.9	493.1 ± 116.1	612.7 ± 100.8	591.7 ± 122.9
31–40	228.8 ± 48.3	204.4 ± 42.1	278.4 ± 74.9	257.9 ± 61.4	438.8 ± 112.6	422.0 ± 104.1	492.1 ± 129.9	496.1 ± 112.2	501.9 ± 113.6	465.6 ± 101.7	576.7 ± 133.1	569.7 ± 107.7

**Table 2:** Comparison of mean CT values within the ROI between different trajectories in male and female

Age group	Traditional pedicle trajectory		Cortical bone trajectory		Modified cortical bone trajectory	
	Male	Female	Male	Female	Male	Female
21–30	247.0 ± 59.0	318.8 ± 69.4	438.9 ± 100.4	520.4 ± 105.4	502.1 ± 102.7	602.2 ± 109.8
31–40	216.6 ± 46.4	268.1 ± 68.4	430.4 ± 107.4	494.1 ± 119.8	483.7 ± 108.0	573.2 ± 119.6

**Table 3:** Comparison of the length between the CBT and MCBT

Length	Cortical bone trajectory	Modified cortical bone trajectory
	34.6 ± 0.69	39.1 ± 1.02

## Discussion

Traditional trajectory (TT) technique was the most common form of posterior fixation for lumbar spine fusion surgery. In the current rapidly growing aging population and the number of patients suffering from spinal diseases, posterior fixation of the lumbar spine in patients with osteoporosis still faces challenges [21, 22], and complications were the common problems [23]. In order to provide the superior fixation for patients, various attempts have been made by domestic and foreign scholars from the design of screw shape to screw track consolidating. However, expandable screws and hydroxyapatite-coated screws were expensive and cannot be widely used [24, 25], while bone cement was expensive and had certain complications and safety risks. Since cortical bone does not deform and degenerate significantly with the aging [26], the basis for the proposal and clinical application of CBT has been laid.

### Biomechanical properties of the MCBT

Previously, several finite element analysis studies were conducted by our team. MCBT effectively avoided the facet joint violation, stress tolerance, and fixation stability was superior than the CBT and traditional technique [11]. Hybrid fixation techniques of MCBT and traditional trajectory at the L4–L5 segment provided superior stability compared to the single fixation system using the same trajectory craniocaudally [13]. The ideal diameter for the L4 vertebral body using MCBT is 5.0 mm, in addition, the pullout strength and stability of the screw were improved significantly [12]. Compared with CBT, the volume of stress area and stress concentration point of MCBT were significantly reduced [14].

### Comparison of screw track bone density

Some scholars use the Hounsfield Units (HU) of CT images for bone mineral density (BMD) and bone strength positively correlated with dual-energy X-ray (DEXA), and HU assessment has the advantage of being reliable, convenient, and affordable, and also predicts postoperative screw loosening and pedicle screw pull-out strength in the lumbar spine [27–30]. The most important risk factor for postoperative screw loosening is osteoporosis [23]. The main fixed are of the pedicle screw was cancellous bone. When osteoporosis occurs, for every 10 g/cm^3^ decrease in bone density, the maximum screw withdrawal resistance decreases by 60 N [31].

Related studies have confirmed that the HU of CBT screw track was greater than that of TT. Jin et al. [18] found that CBT is 1.7–2.3 times higher than TT, zhang et al. [19] found that CBT is 1 times higher than TT, and Kojima et al. [20] found that CBT is 4 times higher than TT, mainly because of the high cortical bone content in the travel area of CBT. The above findings in the literature are consistent with our findings, and the comparative CT values of the screw tracks in this study resulted in MCBT > CBT > TT, and the comparisons between the groups were statistically significant.

Reviewing the anatomy of the lumbar spine, the thickness of the bony cortex around the pedicle varies as follows: inferior wall > superior wall > medial wall > lateral wall [32], with the cortical bone of the endplate being thinner in the central zone and thicker in the marginal zone at the junction with the cortical bone of the vertebral body [33]. In traditional CBT, the screw entry point was closer to the lateral wall of the arch, which was still distant from the medial wall of the arch, where the bone cortex was relatively thicker [16, 17]. The MCBT technique moves the screw entry point 2.0–3.0 mm toward the midline based on CBT, which not only increases the bone thickness around the screw placement point and the holding force with the medial wall of the arch, but also reduces the incision exposure during screw placement. In addition to the changes in the screw entry point, the MCBT technique further increases the contact between the screw and the cortical bone around the screw path, especially the bone cortex of the medial wall of the pedicle and the lateral edge of the upper endplate of the vertebral body, by increasing the abduction angle and decreasing the cephalic tilt angle, based on the 10° of screw abduction angle of the traditional CBT technique. It further improves the biomechanical properties [7, 8, 34].

The results of this study further confirms that the bone density of the MCBT is significantly greater than that of CBT.

The CT value of the screw track was positively correlated with the pull-out strength of the pedicle screw [30]. Ueno et al. [35] found that the pull-out strength of CBT was greater than that of TT, regardless of the type of screw. CBT increases the uniaxial yield pullout load by 30% and 1.7 times higher torque than TT screw [4, 5]. Our finding was that MCBT screw also had better pullout resistance than TT screw [12]. By comparing the anatomical characteristics of the CBT and MCBT, and by combining the mean CT values of the MCBT screw track with those of the CBT and statistically significant (P < 0.05). We concluded that the pullout resistance of MCBT screw is mainly attributed to the superior qualities of the screw track.

### Length of the screw path

**Fig. 3:**
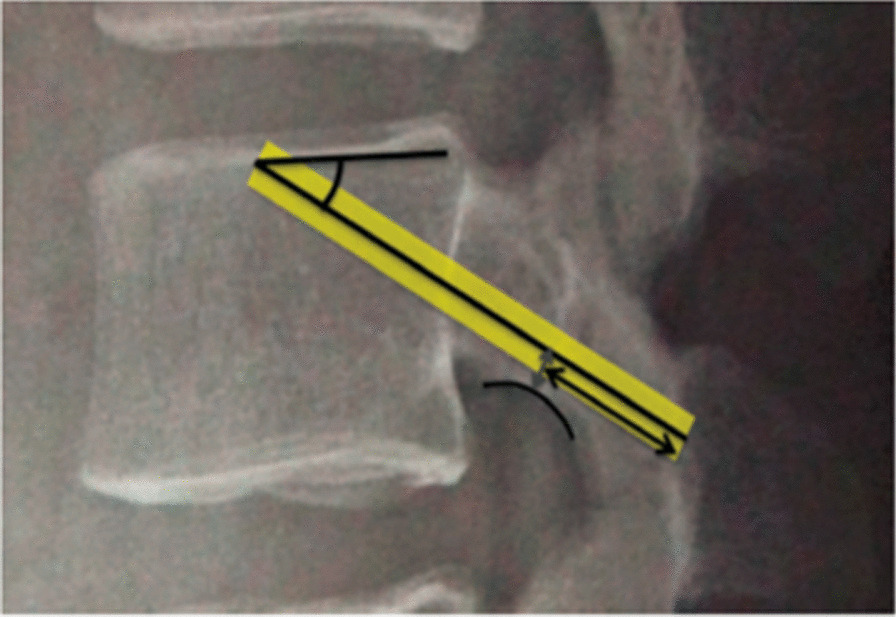
Length of the CBT screw within the vertebral body and the total length [16]

The fixation strength of posterior fixation was not only related to the strength of the bone, but also to the length of the screw [36]. Varghese et al. [37] confirmed through biomechanical study that the torque of the screw during insertion into the bone is the best predictor of the final bone-screw interface failure. However, it is worth noting that the most important factor affecting the insertion torque of CBT was the screw length within the lamina, independent of the length within the vertebra or the total screw length (Fig. 3) [17]. We believe that this conclusion was reached precisely because of the inherent defects of the traditional CBT and the failure to effectively utilize the cortical bone throughout the screw trajectory, especially the medial and inferior wall of the pedicle and the bony cortex at the lateral edge of the upper endplate of the vertebral body [8, 9]. Fujiwara et al. [34] found that the insertion torque of the CBT screw was positively correlated with screw length, negatively correlated with the distance between the screw and the medial wall of the pedicle and the distance from the screw to the superior endplate. Concept of MCBT placement with longer screw tracks and closer to the medial wall of the pedicle was also in line with this findings.

**Fig. 4:**
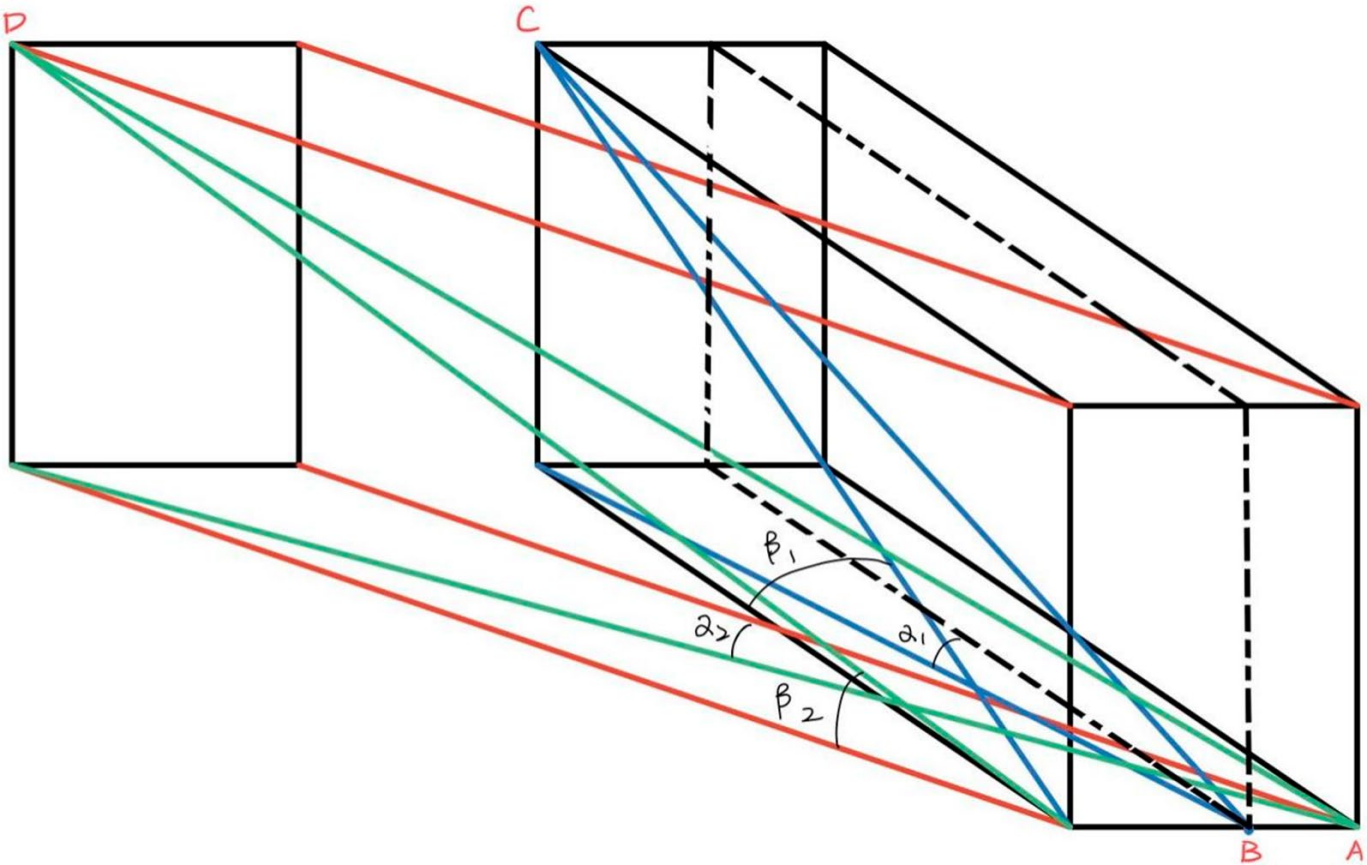
Spatial imaginary map of CBT and MCBT screw path; BC: CBT, α1: the CBT cross-sectional abduction angle, β1: the CBT cross-sectional abduction angle, AD: MCBT, α2: the MCBT cross-sectional abduction angle, β2: the MCBT cross-sectional abduction angle

Chen et al. [38] demonstrated the anatomical parameters of CBT screw placement proper to the Chinese adults. The common length specification of screws in Chinese adult lumbar traditional CBT technique was 35–40 mm, the upper limit of screw diameter safety at each level was L1: 5.5 mm, L2: 5.5–6.0 mm, L3: 6.5–7.0 mm, L4: 7.5 mm, L5: 8.0 mm, the average abduction angle of screw placement at each level of lumbar vertebra was 9.20° ± 2.11°, and the average head inclination angle was 26.41° ± 4.22°. The abduction angle of the MCBT screw placement technique was 10° in the L1 and L2 vertebral bodies and 15° in the L3 vertebral body, while the L4 and L5 vertebral bodies were 15°–20°, with a smaller head inclination angle than CBT, and the distance this trajectory traveled in the lumbar spine [9]. As showing in the Fig. 4, AD > BC, a1 < a2, β1 > β2. This corresponds to the results of our study and other paper [34], in the comparison of the length of the screw track, the length of the MCBT in each group was greater than that of CBT, and it was statistically significant.

However, It is difficult to insert the MCBT screw with freehand in high accuracy because it has a subtle difference from CBT including the difference of several millimeters between the entry points of the two trajectories and few degrees between the mediolateral and craniocaudal angles. Penner et al. [39] obtained the comparable with the higher accuracy of CBT reported for the robot-assisted pedicle screw placement using preoperative 3D CT planning. 3D printed navigation template was investigated by our team previously and demonstrated that it improves the accuracy of the MCBT screw placement [40]. Currently, CBT screws combined with robotic screw placement have become a safe and effective method with the advantages of precise screw insertion and minimal trauma [41, 42]. In addition, emerging artificial intelligence (AI) technology plays an important role in solving many problems faced in spine surgery [43]. We strongly believe that the MCBT technique will be perfectly combined with robotics and AI in the near future.

In addition, McLachlin et al. [44] found the “teeter-totter” phenomenon by CT reconstruction of the loosened TT, early loosened screw makes use of the cortical bone as the fixation point, with the anterior head swinging in the cancelous bone. MCBT due to the extension of the screw path, the head of the screw reaches the lateral edge of the upper endplate of the vertebral body, while the entry point of the MCBT close to the thicker medial wall of the pedicle, so that the screw head and tail fixed which reduces the incidence of the “teeter-totter” phenomenon, screw loosening, and postoperative revision rate. According to the study above, combined with the anatomical basis of the MCBT and the results of our study, the MCBT screw with longer tract has better extraction resistance than CBT to a certain extent.

### Limitations

Certain limitations were inherent in the present study: First, the sample size was not enough. Second, size of the screws selected was fixed, screw with different diameters and lengths were not analyzed. Third, only the patients with normal bone density were analyzed. Anatomical characteristics of vertebral body and changes with age will be discussed in the future.

## Conclusion

The results of the comparison of the CT values of the three screw tracks were: MCBT > CBT > TT, and the MCBT screw track had superior strength of the bone-screw surface. The MCBT screw track was longer than the CBT, and it was easier to reach the anterior column of the vertebral body to achieve three-column fixation of the spine. The MCBT technique, with less surgical exposure and better biomechanical properties, may become a new approach in spine surgery.

## Data Availability

Data used to support the findings of this study are included within the article.
